# Phosphonate-based iron complex for a cost-effective and long cycling aqueous iron redox flow battery

**DOI:** 10.1038/s41467-024-45862-3

**Published:** 2024-03-25

**Authors:** Gabriel S. Nambafu, Aaron M. Hollas, Shuyuan Zhang, Peter S. Rice, Daria Boglaienko, John L. Fulton, Miller Li, Qian Huang, Yu Zhu, David M. Reed, Vincent L. Sprenkle, Guosheng Li

**Affiliations:** 1 Energy & Environment Directorate, Pacific Northwest National Laboratory, Richland, WA 99354 USA; 2 School of Polymer Science and Polymer Engineering, The University of Akron, Akron, OH 44325 USA; 3 Physical & Computational Science, Directorate, Pacific Northwest National Laboratory, Richland, WA 99354 USA

**Keywords:** Batteries, Energy science and technology

## Abstract

A promising metal-organic complex, iron (Fe)-NTMPA_2_, consisting of Fe(III) chloride and nitrilotri-(methylphosphonic acid) (NTMPA), is designed for use in aqueous iron redox flow batteries. A full-cell testing, where a concentrated Fe-NTMPA_2_ anolyte (0.67 M) is paired with a Fe-CN catholyte, demonstrates exceptional cycling stability over 1000 charge/discharge cycles, and noteworthy performances, including 96% capacity utilization, a minimal capacity fade rate of 0.0013% per cycle (1.3% over 1,000 cycles), high Coulombic efficiency and energy efficiency near 100% and 87%, respectively, all achieved under a current density of 20 mA·cm^-^². Furthermore, density functional theory unveils two potential coordination structures for Fe-NTMPA_2_ complexes, improving the understanding between the ligand coordination environment and electron transfer kinetics. When paired with a high redox potential Fe-Dcbpy/CN catholyte, 2,2′-bipyridine-4,4′-dicarboxylic (Dcbpy) acid and cyanide (CN) ligands, Fe-NTMPA_2_ demonstrates a notably elevated cell voltage of 1 V, enabling a practical energy density of up to 9 Wh/L.

## Abstract (web summary)

Here, authors report an iron flow battery, using earth-abundant materials like iron, ammonia, and phosphorous acid. This work offers a solution to reduce materials cost and extend cycle life in energy storage applications for grid decarbonization.

## Introduction

In response to the urgent need to address global warming and climate change, there has been a surge in movements and campaigns within both the civil and technological sectors aimed at facilitating the transition from fossil fuels to sustainable and clean energy sources^1–4^. One of the key strategies to address this critical issue is the development of efficient, cost-effective, and reliable energy storage technologies that can support and stabilize intermittent renewable energy sources like wind and solar systems^5–7^. Among the various available battery energy storage systems, redox flow battery (RFB) technology stands out as a promising solution in this endeavor, which offers important features including superior safety, durability, scalability, decoupled power/capacity characteristics, and the potential for cost-effectiveness^8–10^. Specifically, vanadium redox flow batteries (VRFBs), which represent the most popular and mature technology among RFBs, leverage the distinctive property of vanadium existing in four different oxidation states (V^2+^ and V^3+^ for the anolyte, and VO^2+^ and VO_2_^+^ for the catholyte). This unique utilization of vanadium in both electrolytes serves to mitigate the performance degradation induced by crossover effects in the RFBs^11^. It is important to highlight that VRFB systems, being a highly promising energy storage technology, have been frequently showcased in large-scale demonstrations in recent years^12–14^.

Nonetheless, the materials scarcity and price volatility of vanadium underscore the pressing need to develop technologies that go beyond-VRFBs^15^. Among various reported redox chemistries using Zn^16–19^, Cr^20,21^, aqueous soluble organics^22–27^ in RFBs, iron redox flow battery (Fe-RFB) shows the advantage of using resource abundant Fe as the redox active material in its electrolytes^28^. Conventional Fe-RFB, which has been invented and developed since the early 1980s, consists of two redox couples, Fe/Fe(II) and Fe(II)/Fe(III) as shown below, for anolyte and catholyte, respectively^29^.

1 $${{{{{\rm{Anode}}}}}}: \qquad 2{{{{{\rm{Fe}}}}}}\to 2{{{{{\rm{Fe}}}}}}\,\left({{{{{\rm{II}}}}}}\right)+2{{{{{{\rm{e}}}}}}}^{-}\qquad\quad{{{{{\rm{{E}}}}}}}_{0}=-0.44\,{{{{{\rm{V}}}}}}\;{{{{{\rm{vs}}}}}}.\,{{{{{\rm{SHE}}}}}}$$

2 $${{{{{\rm{Cathode}}}}}}:\qquad2{{{{{\rm{Fe}}}}}}\,\left({{{{{\rm{III}}}}}}\right)+2{{{{{{\rm{e}}}}}}}^{-}\to 2{{{{{\rm{Fe}}}}}}\,\left({{{{{\rm{II}}}}}}\right)\quad\quad\quad{{{{{\rm{E}}}}}}_{0}=0.77\,{{{{{\rm{V}}}}}}\; {{{{{\rm{vs}}}}}}. \,{{{{{\rm{SHE}}}}}}$$

3 $${{{{{\rm{Overall}}}}}}:\qquad{{{{{\rm{Fe}}}}}}+2{{{{{\rm{Fe}}}}}}\,\left({{{{{\rm{III}}}}}}\right)\to 2{{{{{\rm{Fe}}}}}}\,\left({{{{{\rm{II}}}}}}\right)\quad\quad{{{{{\rm{E}}}}}}_{0}=1.21\,{{{{{\rm{V}}}}}}$$

From the above redox reactions, one can find that the anode redox reaction of conventional Fe-RFB involves the use of Fe plating/stripping, in contrast to catholytes consisting of solvated aqueous [Fe(II)·(H_2_O)_6_]^2+^/[Fe(III)·(H_2_O)_6_]^3+^ complexes^30,31^. In this sense, the conventional Fe-RFB can be defined as a hybrid-type flow battery, where the power and capacity are not completely decoupled and thus poses some challenges, such as limited duration, concerns about possible dendrite formation from repeated stripping/deposition processes on Fe anode, etc. One alternative route for replacing the Fe anode in the conventional Fe-RFB to realize all soluble electrolytes involves the use of soluble anolytes including Cr(II)/Cr(III), and V(II)/V(III) electrolytes, etc., which are known as iron chromium (FeCr)-RFBs^21,32–34^ or iron vanadium (FeV)-RFBs^35^.

Another possible approach is to employ a soluble Fe anolyte instead of using Fe plating to achieve all soluble Fe-RFB; this may seem quite counterintuitive because Fe ions exist mostly in two valence states (such as Fe(II), Fe(III)), which are already used in the catholyte. However, it is important to note that Fe(II) or Fe(III) ions are both capable of forming stable complexes via coordinating with various ligands, as shown in Eq. (4). By combining two equations Eqs. (2) and (4), the redox potential of the Fe complexes can be formulated as Eq. (5)^36^ (see Supplementary Information Note 1 for more details).

4 $${{{{{\rm{Fe}}}}}}({{{{{\rm{III}}}}}}){{\cdot }}{{{{{{\rm{L}}}}}}}_{{{{{{\rm{x}}}}}}}+{{{{{{\rm{e}}}}}}}^{-}\to {{{{{\rm{Fe}}}}}}({{{{{\rm{II}}}}}}){{\cdot }}{{{{{{\rm{L}}}}}}}_{{{{{{\rm{x}}}}}}}$$

5 $${{{E}}}_{{{{{{\rm{Fe}}}}}}({{{{{\rm{III}}}}}}){{\cdot {{{{{\rm{Lx}}}}}}}}/{{{{{\rm{Fe}}}}}}({{{{{\rm{II}}}}}}){{\cdot {{{{{\rm{Lx}}}}}}}}}^{0}={{{E}}}_{{{{{{\rm{Fe}}}}}}({{{{{\rm{III}}}}}})/{{{{{\rm{Fe}}}}}}({{{{{\rm{II}}}}}})}^{0}+R{{\cdot }}T/F{{\cdot }}{{{{\mathrm{ln}}}}}\left[{K}_{{{{{{\rm{Fe}}}}}}({{{{{\rm{II}}}}}})\cdot {{{{{\rm{L}}}}}}x}/{K}_{{{{{{\rm{Fe}}}}}}({{{{{\rm{III}}}}}}){{\cdot }}{{\rm L}}x}\right]$$

Where, *E*^0^_Fe(III)·L*x*/Fe(II)·L*x*_ is the redox potential of the Fe complexes, *E*^0^_Fe(III)/Fe(II)_ is the standard redox potential of Fe(III)/Fe(II) (0.77 V vs. SHE), and *K*_Fe(II)·L*x*_ and *K*_Fe(III)·L*x*_ are equilibrium/formation constants for Fe(II) and Fe(III) complexes, and L*x* is ligand complexing with Fe ions, respectively.

**Fig. 1:**
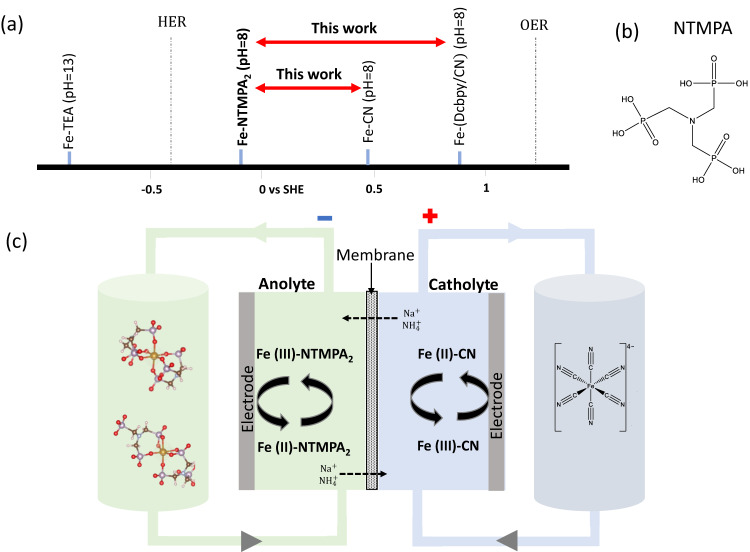
Aqueous Fe redox flow battery in this work. **a** Redox potentials of representative Fe complexes vs. SHE, along with hydrogen evolution reaction (HER) and oxygen evolution reaction (OER) potentials at acidic conditions with the consideration of the overpotentials on carbon electrode, are briefly compared. **b** Molecular structure of NTMPA, nitrogenous triphosphonate, nitrilotri(methylphosphonic acid). **c** Schematic view of Fe-RFB consisting of Fe-NTMPA_2_ anolyte and Fe-CN catholyte.

The implication of Eq. (5) is quite simple and fascinating because it tells that the redox potential of Fe(II) and Fe(III) complexes can be altered from its standard redox potential, *E*^0^_Fe(III)/Fe(II)_, by changing the ligands bound to them^37,38^. For example, a Fe complex with a ligand that has a larger Fe(II) equilibrium constant than the Fe(III) can shift the redox potential upward, and vice versa for a different ligand with a larger Fe(III) equilibrium constant, the redox potential can be shifted downward. Therefore, from a practical point of view, Eq. (5) indicates that both the anolyte and catholyte of Fe-RFB can be synthesized from Fe(II)/Fe(III) complexes with different ligands as long as two different Fe complex pairs provide appropriate potential difference, which will ultimately represent the output voltage of the Fe-RFB. The redox potentials of several representative Fe(II)/Fe(III) anionic complexes are listed in Fig. 1a.

The Fe complexes with more positive potential are suitable as the catholyte, and those with more negative potentials as the anolyte. With ferrocyanide (Fe(CN)_6_^4−^or Fe-CN)^39–41^ being one of the popular redox materials for the Fe catholytes (0.37 V vs. SHE) as a potential low cost and stable redox material, while research on feasible Fe complexes for the anolyte in aqueous Fe-RFB has been stagnate due to the challenges of designing suitable Fe complexes. Fe-triethanolamine (TEA) complex reported by Gong et al.^42^ is one of the few candidates for Fe anolytes with exceptionally high negative redox potential. However, Fe-TEA based anolytes face degradation issues due to their high pH, Fe plating, and the crossover of free TEA, significantly impacting their stability. A recent study involving the modification of TEA using sulfonic moiety (DIPSO) has demonstrated more stable cathodic behaviors, and the capacity decay rate was reported as 0.12%/cycle^43^. Moreso, metal complex based anolytes for application in near pH-neutral RFB are very limited, as most anolytes developed are employed in acid RFBs to prevent the precipitation of Fe(II)/Fe(III) hydroxide. It’s worth emphasizing that a pH-neutral electrolyte is particularly advantageous because it mitigates base/acid-catalyzed degradation of the redox active materials and minimizes side reactions, such as oxygen and hydrogen evolution reactions, which are often encountered in alkaline and acidic electrolytes^44,45^. Therefore, it is highly desirable to strategically design/synthesize novel Fe complexes, which has reasonably negative redox potential vs. Fe-CN complex, assuming Fe-CN as the catholyte, and present good solubility and stability at near neutral or slightly basic pH.

Here, we report a new Fe complex synthesized using nitrogenous triphosphonate, nitrilotri(methylphosphonic acid) or NTMPA shown in Fig. 1b, as a complexing ligand for the application in near-neutral pH (pH = 8) aqueous all soluble Fe-RFB application. NTMPA based Fe complex presents attractive properties such as highly reversible redox reaction, excellent cyclability (0.0012%/cycle), high solubility and cost-effectiveness, which render it a compelling choice for Fe-RFBs. Figure 1c shows a schematic representation of Fe-NTMPA_2_/Fe-CN aqueous RFB, with the corresponding redox reaction during the charge and discharge process. High cell voltage of around ~1 V was achieved when Fe-NTMPA_2_ was paired with high redox potential catholyte, iron- 2,2′-bipyridine-4,4′-dicarboxylic (Dcbpy) acid and cyanide complex, Na_4_[Fe(Dcbpy)_2_(CN)_2_] (Fe-Dcbpy/CN). The application of phosphonate-based ligands in all-soluble Fe-RFBs marks a significant milestone in the advancement of beyond-VRFB technologies, bringing RFB technology one step closer to achieve cost-effective Fe-RFBs for practical applications.

## Results and discussion

**Fig. 2:**
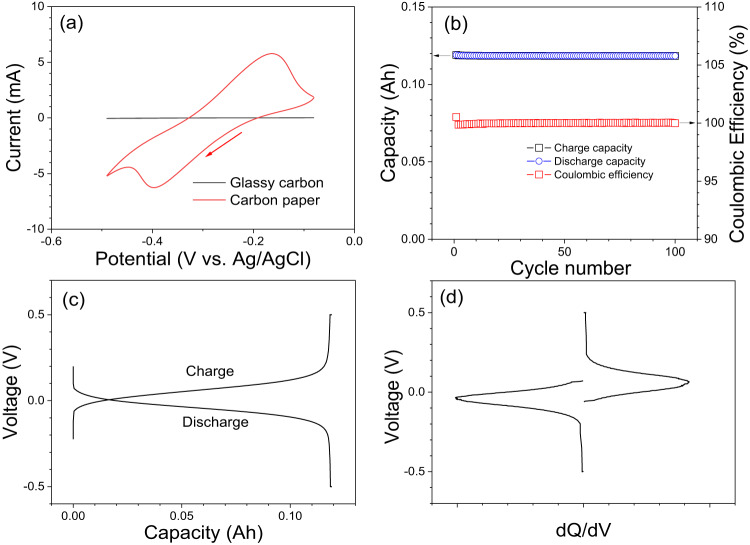
Redox potential and symmetric cell test. **a** Cyclic voltammograms of 0.67 M Fe-NTMPA_2_ complex electrolyte recorded at a scan rate of 2.5 mV·s^−1^ with two different working electrodes, glassy carbon (black) and carbon paper (red). **b** Symmetric cell testing results of 0.67 M Fe-NTMPA_2_ as both catholyte and anolyte for 100 cycles at a current density of 20 mA·cm^−2^. **c** Typical voltage profile and (**d**) differential capacity analysis for the symmetric cell test.

First, the Fe(III)-NTMPA_2_ (Fe-NTMPA_2_) complex was prepared with Fe to ligand optimal ratio of 1:2 and its electrochemical properties evaluated with cyclic voltammetry (CV). CV studies of 0.67 M Fe-NTMPA_2_ solution displayed one reversible redox couple with oxidation and reduction peaks in a redox potential range of −0.40 and −0.17 V at 2.5 mV/s scan rate (red in Fig. 2a), resulting in a half redox potential of near −0.30 V vs. Ag/AgCl reference electrode. CV measurements for the Fe-NTMPA_2_ anolytes at higher scan rates of 5, 7.5, 9, 10 and 11 mV·s^−1^ are also shown in Fig. S1. It’s worth noting that CV scans conducted with a conventional glass carbon electrode (1 mm diameter) did not exhibit any meaningful cathodic or anodic current responses, as indicated by the black line in Fig. 2a. In contrast, a pair of redox peaks were clearly observed when using a custom-made working electrode crafted in the lab using carbon paper (see the experimental section, Fig. S2 and caption).

An initial battery evaluation was conducted using a symmetric flow cell test, where 7 mL of 0.67 M Fe-NTMPA_2_ electrolyte (Theo. capacity of 125 mAh) served as both the catholyte and anolyte to determine the stability of this complex and ascertain its suitability as a redox active material in RFB. The symmetric flow cell test is carried out with a current of 200 mA (20 mA·cm^−2^, ~1.7 C) for 100 cycles (~120 h). Notably, the capacity plot shown in Fig. 2b consistently registers at 119 mAh (96% capacity utilization) with a CE of ~100% and reveals no degradation over the course of 100 cycles. The typical voltage profiles of the symmetric cell testing in Fig. 2c show quite smooth and single plateau, and a differential capacity (dQ/dV vs. V, Fig. 2d) plot also supports the single-phase reaction for Fe-NTMPA_2_ electrolytes during the charge and discharge processes.

**Fig. 3:**
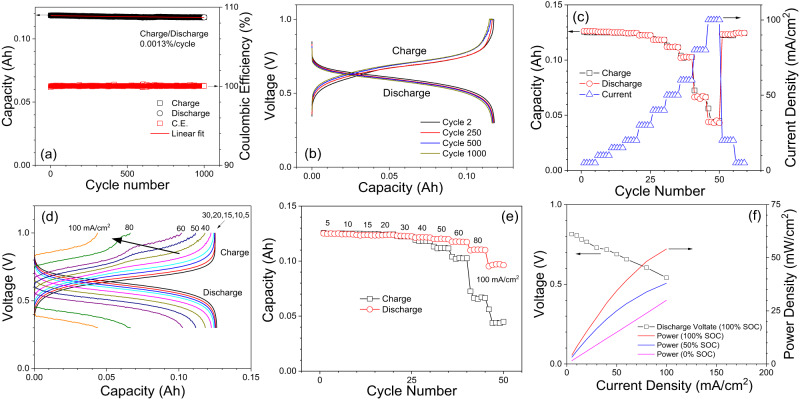
Cell test for aqueous Fe redox flow battery. **a** Full cell test of 0.67 M Fe-NTMPA_2_ anolyte paired with 0.67 M Fe-CN catholyte at 20 mA·cm^−2^ for 1000 cycles. Capacity and Coulombic efficiency (C.E.) plots vs. cycle number. **b** Comparison for the representative charge and discharge voltage profiles over 1000 cycles. **c** Capacity response vs. different current densities (5–100 mA·cm^−2^). **d** The charging/discharging voltage profiles for the different currents densities (5–100 mA·cm^−2^). Black arrow indicates voltage set-ups appeared for higher current densities. **e** Capacity response from asymmetric current densities for the charge and discharge, respectively. For asymmetric charge (black), the discharging current is fixed at 20 mA·cm^−2^, while the charging current increases from 5–100 mA·cm^−2^. Conversely, for asymmetric discharging (red), the charging current remains fixed at 20 mA·cm^−2^ while the discharging current varies in the same range. **f** Cell discharge voltage (100% SOC) and discharging power density plots vs. current density. Discharging power densities are shown for the depth-of-discharge (DoD) at 0, 50 and 100%, respectively.

Following the CVs and symmetric cell tests, a full cell test comprising both galvanostatic and potentiostatic processes (see experimental section for specific details) was conducted. This test employed balanced electrolytes, consisting of 7 mL of 0.67 M Fe-NTMPA_2_ (with a theoretical capacity of 125 mAh) as the anolyte and a 0.67 M Fe-CN aqueous solution (with a theoretical capacity of 125 mAh) as the catholyte. Figure 3a shows overall charge/discharge capacity trend over 1000 cycles at a current density of 20 mA·cm^−2^ (200 mA), which spanned about 50 days. Notably, from a simple linear fitting, the charge/discharge capacity decay rate calculated for 1000 cycles is about 0.0013%/cycle, which indicates that this Fe-RFB loses about 1.3% its capacity every 1000 cycles. Specifically, the reported capacity degradation rate for Fe-NTMPA_2_ is two orders of magnitude lower than that (0.12% per cycle) reported for TEA and its analogues. The superior cyclability can be attributed to the benign pH and mild redox potential, which facilitate the excellent stability of the Fe-NTMPA_2_ complex, preventing parasitic reactions including decomposition of Fe complex and high-pH induced SOC imbalance of the ferrocyanide catholyte. Coulombic efficiency remains consistently close to 100% throughout the entire 1000 cycles. It’s worth noting that the energy efficiency of this Fe-RFB experiences a slight decline, from 87% to 80% after 1000 cycles, contrasting with the stable CE. As shown in Fig. 3b, this decline in energy efficiency is due to the gradual increase in overpotential and the lengthening of the potentiostatic charge/discharge period observed throughout the 1000 cycles. The slight increase in overpotential could be attributed to the reduction of active surface area or flow rate due to electrode clogging caused by peristaltic tube wear (see Fig. S3 and its figure caption). Battery tests at different current densities varying from 5 to 100 mA·cm^−2^ were examined as shown in Fig. 3c. As observed, the capacity utilization remains consistently high until the current density exceeds approximately 30 mA·cm^−2^. Beyond this point, the rate of capacity decline accelerates as the current density is further increased. It should be noted that the test fully restores to its full capacity when the current decreases to lower values. This observation further supports that the temporary decrease in capacity at higher currents is attributed to the kinetic limit and not to any parasitic loss; otherwise, it would result in a permanent capacity loss. The charging voltage profiles under different current densities reveal an intriguing phenomenon—a noticeable voltage jump (indicated by a black arrow in Fig. 3d) occurs when the current density exceeds ~30 mA·cm^−2^. However, it’s worth noting that a corresponding voltage plateau does not appear in the discharging voltage profiles, which show smooth curves with a single plateau. To rule out possible contributions from ferrocyanide (Fe-CN) catholytes to the observed voltage jump, we conducted symmetric cell testing. In this experiment, Fe-NTMPA_2_ electrolytes at 50% SOC were utilized as both the catholyte and anolyte, with varying current densities for charge and discharge. As anticipated, a similar voltage jump was observed at high SOCs, as shown in Fig. S4, for symmetric (Fe-NTMPA_2_/Fe-NTMPA_2_) cells. We attribute this phenomenon to the charge kinetics and inherent characteristics of the Fe-NTMPA_2_ complex rather than from Fe-CN catholytes. Figure 3e presents the results from two sets of what are commonly referred to as asymmetric charge/discharge tests. An asymmetric charge (black in Fig. 3e) is done by fixing the discharging current at 20 mA·cm^−2^ while varying the charging current from 5 to 100 mA·cm^−2^. Similarly, the asymmetric discharge (red in Fig. 3e) is done by fixing the charging current at 20 mA·cm^−2^ while varying the discharging current. The results shown in Fig. 3e clearly shows a higher capacity retention for the asymmetric discharge, which indicates a faster discharge kinetic compared to the charge process. Further elaboration on this matter can be found in the subsequent section, where we correlate these findings with the results obtained from DFT calculations. Figure 3f displays the discharge capabilities of this RFB at three different depth-of-discharges (DODs), showing an output power density of 60 mW·cm^−2^ (0% DOD) at a current density of 150 mA·cm^−2^. In addition, we performed a simple test in which the battery was kept in a fully charged state for an extended period before commencing the discharge cycle. Preliminary results indicate >99% discharge (Fig. S5) after maintaining this Fe-RFB at 100% SOC over 7 days, demonstrating minimal self-discharge at high SOC.

**Fig. 4:**
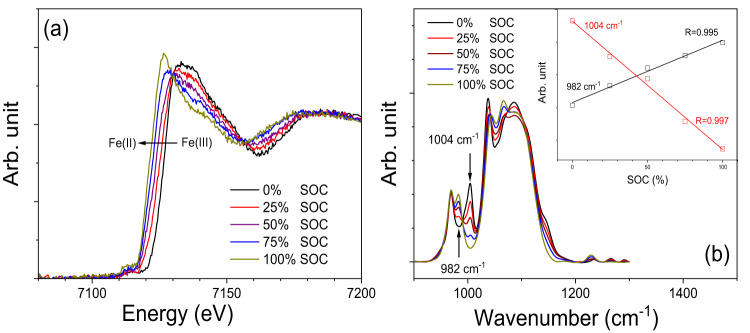
Spectroscopic characterization for Fe-NTMPA_2_ anolytes. **a** Spectra from X-ray absorption near-edge spectroscopy (XANES) and (**b**) Attenuated Total Reflectance (ATR) spectra, for Fe-NTPMA_2_ anolytes obtained at different SOCs (0, 25, 50, 75, and 100%). The insert of (**b**) shows the linear fits for the intensities of 982 and 1004 cm^−1^ peaks vs. SOCs.

Figure 4a presents ex-situ x-ray absorption spectroscopy (XAS) measurements, utilizing Fe K-edge XAS to determine the oxidation states of Fe through XANES edges. These measurements were carried out on the Fe-NTMPA_2_ anolytes acquired at different SOCs, ranging from 0% to 100% SOC. Clearly, XANES peaks shift to the left (lower energy) while SOCs change from 0% SOC (Fe(III)), to 100% SOC (Fe(II)). Moreover, a straightforward linear combination analysis, as illustrated in Fig. S3a, suggests that XANES spectra corresponding to 25%, 50%, and 75% SOCs can be directly fitted by employing XANES spectra from 0% and 100% as reference standards. The calculated SOCs (Table S1) from the linear combination analysis are 28%, 54%, and 79%, respectively, and are in good agreement with the SOCs determined through coulomb counting (cell testing). Attenuated Total Reflectance (ATR) IR spectra of the Fe-NTMPA_2_ anolyte samples at various SOCs are also shown in Fig. 4b, and exhibit several peaks within the 900–1200 cm^−1^ range, which is commonly associated with the symmetric (ν_s_) or asymmetric (ν_as_) stretching mode of the P−O bonds in phosphonate (−PO_3_^2−^)^46^. In particular, there are two prominent peaks at 982 and 1004 cm^−1^ that exhibit a clear correlation with SOCs of the Fe-NTMPA_2_ anolytes. The intensity of the 982 cm^−1^ peak increases as the SOC of the anolytes rises, and conversely, the intensity of the 1004 cm^−1^ peak decreases when the SOC reaches 100%. The stronger complexation of Fe(III)-NTMPA_2_ compared to Fe(II)-NTMPA_2_, driven by the higher charge density of the Fe(III) ion, promotes electron polarization, consequently resulting in an increase in the force constant of the P−O bond (higher vibrational frequency). The observed blue shift of 22 cm^−1^ in the ATR peaks, transitioning from 982 to 1004 cm^−1^, clearly supports the assignment of the 1004 cm^−1^ peak to the coordinated phosphonate moiety of Fe(III)-NTMPA_2_ complex. The intensity plots of the two ATR peaks vs. SOC exhibit remarkably good linear relationships (R > 0.996), as shown in the inset of Fig. 4b. This indicates that the accurate assessment of the SOCs for Fe-NTMPA_2_ anolytes can be achieved through real-time monitoring of these two ATR peaks, if a suitable ATR cell is integrated.

**Fig. 5:**
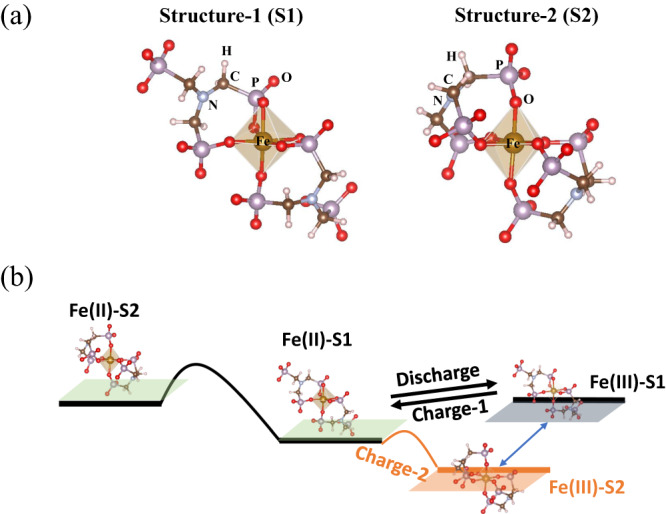
Molecular structures and schematic energy levels for Fe-NTMPA_2_ complexes. **a** Two possible molecular structures of Fe-NTMPA_2_ after density function theory (DFT) geometry optimization. Structure-1 (S1) shows octahedral coordination of Fe ion with two NTMPA ligands, and two phosphonate groups of each NTMPA contribute the first coordination shell. Structure-2(S2) shows a similar octahedral coordination, except all phosphonate groups of each NTMPA involve to the bonds with Fe ion. **b** Schematic view of the redox reaction pathway and the free energy profiles for Fe(II)-NTMPA_2_ and Fe(III))-NTMPA_2_ complexes with different coordination structures.

To gain a deeper insight into the fundamental behaviors of the Fe-NTMPA_2_ complexes in the electrolyte, we have used density functional theory (DFT) to explore several possible bonding configurations of the Fe-NTMPA_2_ complexes. It should be noted that all three phosphonate groups of NTMPA are most likely in a completely deprotonated form (PO_3_^2−^) due to the electrolyte’s pH, which was kept at 8—slightly higher than the reported p*Ka* value of 7.22 for the last deprotonation of phosphonates of the Fe-NTMPA_2_ complexes^46^. Geometry optimization and free energy calculations have been performed in implicit aqueous solvent, with the B3LYP-D3(BJ) exchange correlation functions, yielding two distinct molecular structures (S1 and S2 as shown in Fig. 5a) for the Fe-NTMPA_2_ complexes. While both S1 and S2 exhibit a common distorted octahedral coordination in the first shell for Fe ions, there are substantial differences in the arrangement of the phosphonate groups of the NTMPA ligands within these complexes. From Fig. 5a, we can see that S1 has two phosphonate groups bonded in the octahedral coordination to Fe center of the complex, with two oxygen atoms from one phosphonate group and one oxygen from the other, leaving one unbound phosphonate group from each NTMPA ligand. Conversely, in S2, all three phosphonate groups of the NTMPA ligand each contribute one oxygen atom to the octahedral coordination, in contrast to S1, where there are no unbound phosphonate groups in S2. Upon analyzing the average atomic distances of Fe−O (1.911 Å) and Fe−P (2.853 Å) in S1 and Fe−O (1.883 Å) and Fe−P (3.228 Å) in S2, we observed values that align reasonably well with those obtained from EXAFS analysis for the Fe(II) and Fe(III) complexes (refer to Fig. S6b and Table S2). In the EXAFS analysis, a coordination number of 6 was determined for the first shell of the iron center, indicative of an octahedral coordination geometry. More intriguing findings arise from calculating the differences in free energy between the two optimized structures, S1 and S2, for Fe(II) and Fe(III) complexes. As shown in Fig. 5b and Table S3, there is a relatively large free energy difference between Fe(II)-S2 (higher energy) and Fe(II)-S1(lower energy), which suggests that Fe(II)-NTMPA_2_ complexes are predominantly in Fe(II)-S1 structure at room temperature. On the contrary, DFT calculations suggest that the Fe(III) NTMPA_2_ complex exists in both S1 and S2 structures, as their free energies closely approximate each other. This discrepancy in the favored complex structure between Fe(II)-S1 and Fe(III)-S1/S2 could greatly impact the electron transfer kinetics that play a pivotal role in battery performance. The underlying rationale behind the connection between molecular structure and electron transfer can be succinctly elucidated as follows: During the discharge process, as illustrated in Fig. 5b, Fe(II)-S1 undergoes oxidation to transform into Fe(III)-S1 with relatively little kinetic hindrance (Discharge in Fig. 5b) due to their similar molecular structures. Subsequently, a portion of Fe(III)-S1 converts to Fe(III)-S2. During the charge process, it is highly probable that Fe(III)-S1 first undergoes a reversible redox reaction (Charge-1) at the onset voltage, forming Fe(II)-S1. This is followed by the interconversion between Fe(III)-S2 and Fe(III)-S1, as the direct reduction of Fe(III)-S2 to form Fe(II)-S1 faces a notable energy barrier resulting from ligand reorientation (Charge-2). The reversible redox reaction between Fe(II)-S1 and Fe(III)-S1 might have inherently similar electron transfer kinetics. However, the ligand reorientation process involved in the conversion of Fe(III)-S2 to Fe(III)-S1 could potentially act as a bottleneck for the charging process.

The redox reaction hypothesis described above could have two primary practical impacts for battery performance. First, due to the kinetic bottleneck involved in converting Fe(III)-S2 to Fe(III)-S1, the charging process may exhibit slower kinetics when compared to the discharging process. This postulation aligns well with the asymmetric current testing results in Fig. 3e, where a notably larger decrease in capacity is observed when increasing the charge current compared to when increasing the discharge current. Secondly, despite the presence of a slower kinetic step during the charging process, the battery can still be charged normally with low overpotential if the charging current remains sufficiently low to accommodate this slower kinetics of the interconversion from Fe(III)-S2 to Fe(III)-S1. However, with an increase in charging current to higher levels, it becomes highly likely that the battery will experience large charging overpotentials to trigger the direct reduction (Charge-2) of Fe(III)-S2 to Fe(II)-S1, a process requiring significant ligand reorientation, to sustain the high charging currents. This is also supported by the abrupt voltage increases observed for higher charging currents, as illustrated in Fig. 3d (black arrow).

**Fig. 6:**
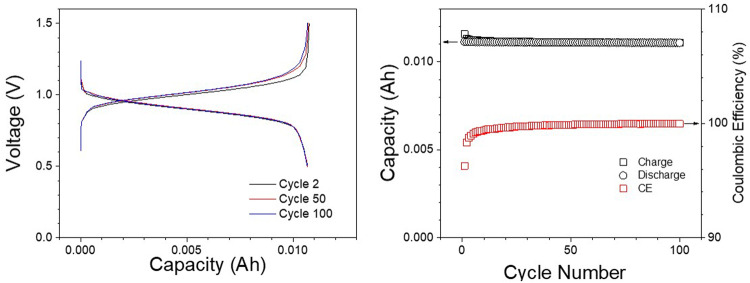
Cell test for Fe-NTMPA_2_ anolytes and Fe-Dcbpy/CN catholytes. **a** Voltage profiles for Fe aqueous flow battery consisting of Fe-NTMPA_2_ anolyte paired with a high redox potential Fe-Dcbpy/CN catholyte. **b** Charge/discharge and Coulombic efficiency plot over 100 cycles.

In the context of practical applications for RFB systems, it’s important to highlight the actual capacity and energy. As detailed in the experimental section, both the Fe-NTMPA_2_ anolyte and Fe-CN catholyte are prepared at a concentration of 0.67 M. This concentration balance results in a theoretical volumetric capacity of ~9 Ah/L with 96% utilization. Despite the relatively low output voltage (~0.6 V) of this Fe-RFB, it demonstrates a specific energy density of ~5 Wh/L when operating at a current density of 20 mA·cm^−2^. One possible approach to increase the energy density is by elevating the potential difference between the anolyte and catholyte, thereby achieving a higher output voltage in the RFB. To demonstrate this strategy, Fe-NTMPA_2_ was paired with a high redox potential catholyte, Fe-Dcbpy/CN ((2,2′-bipyridine-4,4′-dicarboxylic (Dcbpy) acid and cyanide ligands) in Fe-RFBs. This combination exhibited a notably high cell voltage of approximately 1.0 V, which is nearly 0.4 V higher than that achieved with the Fe-CN catholyte. Representative charge/discharge voltage profiles at cycles 2, 50, and 100 are shown in Fig. 6a. Furthermore, the high voltage cell demonstrated a good stability over 100 cycles under a current density of 20 mA·cm^−2^, with a minimal capacity fade and high Coulombic efficiencies (CE) except for a few cycles at the beginning of the test, as shown in Fig. 6b. If one applies the same method to estimate the energy density of this high-voltage Fe-RFB, comprising the Fe-NTMPA_2_ anolyte and Fe-Dcbpy/CN catholyte, the specific energy density could increase from 5 Wh/L to approximately 9 Wh/L. This represents a significantly high practical energy density when compared to existing RFBs that utilize metal-organic coordination complexes.

In addition to the attractive chemical stability and longevity of Fe-NTMPA_2_ complex as an anolyte for all soluble Fe-RFB applications, it is worth emphasizing its practical viability from both the materials supply chain and cost perspective. Fe-NTMPA_2_ mainly consists of FeCl_3_ and NTMPA, which can be synthesized or processed from readily available earth-abundant materials, such as Fe_2_O_3_ (~1$/kg), HCl (~0.01$/kg), ammonia (NH_3_, ~1.1$/kg), formaldehyde (CH_2_O, ~$1/kg), and phosphorous acid (H_3_PO_3_, ~1$/kg), and its synthesis route has been well established for decades^47^. Obtaining a precise cost estimate for the large-scale production of Fe-NTMPA_2_, inclusive of processing costs, is beyond the scope of this initial work. Despite the early stage of exploration, it is undeniable that Fe-NTMPA_2_ holds the potential to be synthesized at a lower cost, which is particularly evident when compared to vanadium electrolytes, leveraging existing infrastructure and materials supply chains.

In summary, a promising metal-organic complex (Fe-NTMPA_2_) comprising of Fe(III) chloride and NTMPA as ligands was synthesized as an electrolyte for near-neutral pH aqueous Fe-RFB system. The redox potential of Fe-NTMPA_2_ is near −0.30 V vs. Ag/AgCl determined by CV measurements, and initial test of symmetric RFBs of Fe-NTMPA_2_ as both the catholyte and anolyte demonstrate robust stability over 100 cycles. Full cell testing employing a high concentration Fe-NTMPA_2_ (0.67 M) as anolyte paired with Fe-CN catholyte showcased a remarkable cycling stability over 1000 consecutive charge/discharge cycles (50 days) with 96% capacity utilization, a minimum capacity fade rate of 0.0013%/cycle (1.3% over 1000 cycles or 0.026%/day), CE of 100% and EE as high as 87% under a current density of 20 mA·cm^−2^. XANES and ATR results affirm that Fe ions from the Fe-NTMPA_2_ complexes are indeed responsible for the redox reaction. Notably, the linear relationship observed between ATR peak intensities and SOCs suggests the possibility for online SOC monitoring by tracking the IR band if an ATR cell is appropriately integrated into the RFB system. DFT calculation unveils two potential coordination structures for Fe-NTMPA_2_ complexes, with the ultimate structure being intricately linked to the valence state of Fe ions. This insight offers opportunities to delve deeper into the ligand coordination environment and electron transfer kinetics within Fe-NTMPA_2_ complexes. The combination of Fe-NTMPA_2_ with a high redox potential Fe-Dcbpy/CN catholyte resulted in a notably higher cell voltage, exceeding that of the Fe-CN catholyte by 0.4 V, and its practical energy density could reach up to 9 Wh/L. Brief assessment of materials cost analysis indicates that Fe-NTMPA_2_ can be synthesized or processed from readily available earth-abundant materials, such as Fe_2_O_3_, HCl, ammonia, formaldehyde, and phosphorous acid through well-established methods. Consequently, adopting phosphonate-based Fe complexes as a general choice for active materials in RFBs could open a viable pathway to alleviate the challenges associated with materials supply chains, which are known to be substantial bottlenecks in many other battery technologies, such as LIB and VRFB, particularly in the context of large-scale energy storage applications.

## Methods

Chemicals and Materials: Iron (III) chloride hexahydrate (Aldrich), ammonium chloride (Alfa Aesar), ammonium hydroxide (Thermo scientific), ammonium phosphate dibasic (J.T. Baker), sodium hydroxide (BTC), sodium chloride (VWR), sodium hydrogen phosphate dibasic, nitrilotri(methylphosphonic acid) (NTMPA, TCI), sodium ferrocyanide decahydrate (Acros Organics), and potassium ferrocyanide trihydrate (Acros Organics) were used as received. Iron-organic complex with 2,2′-bipyridine-4,4′-dicarboxylic (Dcbpy) acid and cyanide ligands, Na_4_[Fe(Dcbpy)_2_(CN)_2_] (Fe-Dcbpy/CN) was synthesized using the previously reported method^48^.

Preparation of Fe-NTMPA_2_ complex: To prepare 50 mL of 0.67 M Fe(III)-NTMPA_2_ (Fe-NTMPA_2_) complex with Fe/NTMPA=1/2 ratio, Fe(III) trichloride hexahydrate was first dissolved in 10 mL water in a beaker and kept under stirring. A solution of 1.34 M NTMPA was placed in a different beaker, and 2.58 M ammonium hydroxide solution added to it and stirred for 30 mins. Thereafter, the NTMPA solution was added slowly using a pipette to the beaker containing Fe(III) trichloride solution. Subsequently, more water and ammonium hydroxide (13.5 M more) were added to dissolve the precipitate formed. Excess water was evaporated to achieve the targeted volume. When necessary, ammonium hydroxide was also added to attain the preferable near-neutral pH. Low-concentration electrolytes were prepared by diluting accordingly the stock solution, namely the 0.67 M Fe-NTMPA_2_ electrolyte.

Preparation of Fe catholytes: 0.67 M ferrocyanide (Fe-CN) catholyte was prepared by dissolving sodium ferrocyanide decahydrate and potassium ferrocyanide trihydrate in water in the ratio of 1:1 (0.335 M each) with ammonium chloride (1.4 M) as added supporting electrolyte. For the Fe-Dcbpy/CN catholyte, the material as obtained was dissolved in water to achieve the desired concentration with 1.4 M sodium chloride employed as the supporting electrolyte.

Electrochemical Characterizations: Cyclic voltammetry was carried out to evaluate the electrochemical properties of the Fe-NTMPA_2_ complex. A three-electrode cell employing a homemade carbon paper piece (5 mm × 25 mm, FreudenbergH23) as the working electrode, glassy carbon rod as the counter electrode and Ag/AgCl reference electrode was utilized. The cyclic voltammograms were recorded using a CH Instruments electrochemical workstation-CHI660E potentiostat across various potential ranges and at different scan rates.

X-ray absorption spectroscopy (XAS): Fe K-edge X-ray absorption near-edge spectroscopy (XANES) and extended X-ray absorption fine structure (EXAFS) data were acquired to assess the oxidation state in liquid Fe-NTMPA_2_ samples and characterize the local coordination environment of Fe. The experimentation utilized a bench-top easyXAFS300 instrument (easyXAFS). For specified states of charges (SOCs), minute quantities of Fe-NTMPA_2_ anolytes were extracted and introduced into a liquid cell designed to handle air-sensitive electrolytes. Subsequently, the liquid cell, housing Fe-NTMPA_2_ anolytes at various SOCs, was inserted into the easyXAFS300 instrument for XAF measurements. Fe K-edge spectra were captured using a Ge(620) spherically-bent crystal analyzer and a Mo anode X-ray tube operating at 25 kV voltage and 20 mA current. Consistent power settings were employed for all spectra, encompassing both sample and background scans. Throughout the experiments, the silicon drift detector deadtime was diligently maintained below 30%.

Attenuated Total Reflectance (ATR) Fourier Transform Infrared (FT-IR): ATR FT-IR spectroscopy measurements were performed using a FT-IR spectrometer (Bruker Vertex 70) equipped with an ATR assembly featuring a diamond crystal. In the case of air-sensitive electrolytes, a liquid dome was applied on top of the diamond crystal to seal the ATR assembly, and the entire sample compartment, including both the ATR assembly and the liquid dome, underwent purging with pure nitrogen gas (oxygen level <1 ppm) to establish an inert atmosphere during the measurements.

Flow battery cell assembly and test: The flow cell was constructed using two electrolyte reservoirs, a cell stack, and a peristaltic pump. The flow frame in the cell stack employed an interdigitated flow field design with an active area of 10 cm^2^. Nafion membrane (NR212) was employed to separate the two half cells. Before use, the NR212 membrane was soaked in ammonium hydroxide or sodium hydroxide solution overnight to convert to NH_4_^+^ or Na^+^ forms. Two carbon papers (2 cm × 5 cm, FreudenbergH23) and 2 ELAT (2 cm × 5 cm, Nuvant) were used as negative electrode, while 3 ELAT (2 cm × 5 cm, Nuvant) were used as positive electrode. The electrolyte containers and the cell stack were connected using Masterflex tubes. A Masterflex L/S peristaltic pump (Cole-Parmer) was used to circulate the electrolytes through cell stack at a flow rate of 40 mL·min^−1^. The flow battery was configured with 7 mL of 0.67 M Fe-NTMPA_2_ as the anolyte and 7 mL of 0.67 M Fe-CN in 1.4 M ${{{{{{\rm{NH}}}}}}}_{4}{{{{{\rm{Cl}}}}}}$ as the catholyte, with (NH_4_)_2_HPO_4_ serving as a buffer in both electrolytes, resulting a theoretical capacity of 125 mAh for both electrolytes. The battery testing was conducted with discharge and charge cut-off voltages of 0.3 and 1.0 V for Fe-NTMPA_2_/Fe-CN, and subsequently increased to 0.5 and 1.5 V for Fe-NTMPA_2_/Fe-Dcbpy/CN electrolytes, respectively. The charging process comprises a galvanostatic charge, e.g. 200 mA (20 mA·cm^−2^), during which the battery is charged to the charge cut-off voltage, followed by a potentiostatic charge-holding phase at the cut-off voltage or constant voltage charge until the current reaches 10 mA. The discharge is done in a same way consisting of galvanostatic discharge and potentionstatic discharge hold until the discharge current reaches 10 mA. Cell testing is conducted using an Arbin BT-200 battery cycler within a N_2_-purged box at room temperature. For the symmetric and asymmetric charge/discharge experiments, the current could vary from 50–1000 mA (5–100 mA·cm^−2^).

Density Functional Theory (DFT): DFT calculations were carried out using ORCA version 4.2.1^49^. The Ahlrichs triple-*ζ* basis set with one set of polarization functions (def2-TZVP)^50^ was used for all calculations. The aqueous solvent environment was taken into consideration on using the universal solvation model (SMD) for water. Geometry optimization and frequency calculations were carried out in implicit aqueous environment using the B3LYP exchange correlation functional with Grimme’s D3 dispersion correction with Becke-Johnson damping. The redox potentials were calculated from the corresponding reaction free energies at 298 K and referenced relative to the standard hydrogen electrode (SHE).

### Supplementary information

Supplementary Information

## Data Availability

Data will be available on request. Supporting figures and more explanation can be found from the Supplementary Information.
