# Supplementary material for: Phosphonate-based iron complex for a cost-effective and long cycling aqueous iron redox flow battery
Source: Nat Commun. 2024 Mar 25;15:2566. doi: 10.1038/s41467-024-45862-3 (PMC10963763; doi:10.1038/s41467-024-45862-3)
Supplement: Supplementary file 1 — Supplementary Information [file 41467_2024_45862_MOESM1_ESM.docx]

**Phosphonate-based Iron Complex for a Cost-Effective and Long Cycling Aqueous Iron Redox Flow Battery**

Gabriel S. Nambafu,^1,†^ Aaron M. Hollas,^1,†^ Shuyuan Zhang,^2^ Peter S. Rice,^3^ Daria Boglaienko,^1^ John L. Fulton, ^3^ Miller Li,^1,‡^ Qian Huang,^1^ Yu Zhu,^2^ David M. Reed,^1^ Vincent L. Sprenkle,^1^ and Guosheng Li^1,*^

^1^ Energy & Environment Directorate, Pacific Northwest National Laboratory, Richland, WA, USA 99354

^2^ School of Polymer Science and Polymer Engineering, The University of Akron, Akron, OH, USA 44325

^3^ Physical & Computational Science, Pacific Northwest National Laboratory, Richland, WA, USA 99354

Corresponding: guosheng.li@pnnl.gov

Supplementary Information Note 1:

Redox potential of Fe(II)/Fe(III) with complexation

Fe(III) + e^−^ → Fe(II) (1) *E*_0_ = 0.77 V vs SHE

Fe(II) + L*_x_* → Fe(II)·L*_x_* (2) *K*_Fe(II)·L_*_x_* = [Fe(II)·L*_x_*]/[Fe(II)]·[L*_x_*]

Fe(III) + L*_x_* → Fe(III)·L*_x_* (3) *K*_Fe(III)·L_*_x_* = [Fe(III)·L*_x_*]/[Fe(III)]·[L*_x_*]

L*x* represents ligand complexing with Fe(II) or Fe(III).

Eq(1) + Eq(2) − Eq(3) will lead to Eq (4) as follow:

Fe (III)·L*_x_* + e^−^ → Fe(II)·L*_x_* (4)

Thus, Δ*G*_4_ = Δ*G*_1_ + Δ*G*_2_ − Δ*G*_3_, where these are corresponding free energies of equations above.

Applying NERNST equation

*E*_4_^0^= Δ*G*_4_/(*Z*·*F*) = (Δ*G*_1_ + Δ*G*_2_ − Δ*G*_3_)/(*Z·F*) where *Z* equals to 1 (one electron reaction) in here and *F* is Faraday constant.

Δ*G*_1_ = *E*_1_^0^ ·*F*, where *E*_1_^0^ = *E*^0^_Fe(III)/Fe(II)_

Δ*G*_2_ = *E*_2_^0^ ·*F*, where *E*_2_^0^= *R*·*T*·ln([Fe(II)·L*_x_*]/[Fe(II)]·[L*_x_*]) = *R*·*T*·ln(*K*_Fe(II)·L_*_x_*)

Δ*G*_3_ = *E*_3_^0^ ·*F*, where *E*_3_^0^= *R*·*T*·ln([Fe(III)·L*_x_*]/[Fe(III)]·[L*_x_*]) = *R*·*T*·ln(*K*_Fe(III)·L_*_x_*)

Therefore,

*E*_4_^0^ = *E*^0^_Fe(III)·L_*_x_*_/Fe(II)·L_*_x_*= *E*^0^_Fe(III)/Fe(II)_ + *R*·*T*/*F*·ln(*K*_Fe(II)·L_*_x_*) − *R*·*T*/*F*·ln(*K*_Fe(III)·L_*_x_*) = *E*^0^_Fe(III)/Fe(II)_ + *R*·*T*/*F*·ln(*K*_Fe(II)·L_*_x_*/*K*_Fe(III)·L_*_x_*)

Figure S1. CVs measurements for 0.67 M Fe-NTMPA_2_ anolytes under different scan rates.


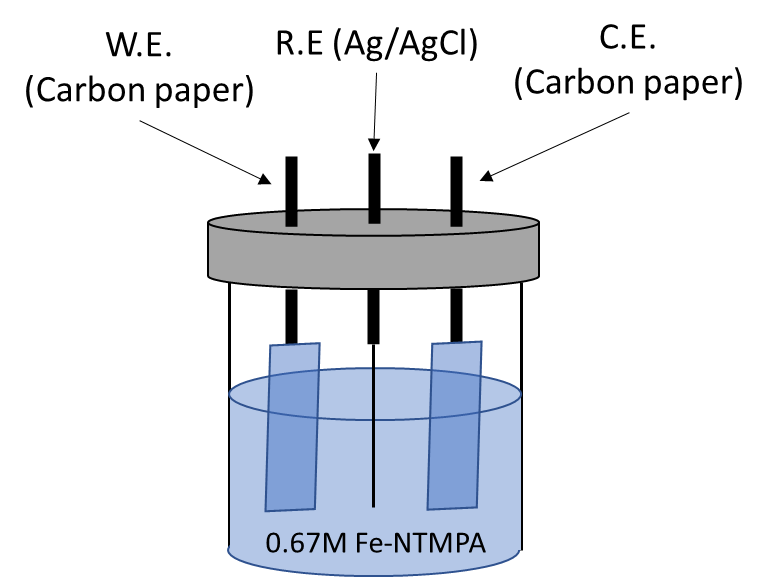


Figure S2. Three-electrodes CV cell. The working electrode is made from carbon paper measuring about 5 mm x 10 mm, and its effective surface area may significantly exceed 50 mm^2^ due to the presence of the high surface area carbon fibers. Initially, the improvement in the performance of the carbon paper electrode is attributed to the increased surface area compared to that of glassy carbon electrode. Carbon paper has a large surface area, thus offering an improved electrochemical activity through increased wettability and providing a large electrochemically active surface area that is available for the redox active materials. It is possible that the surface functional groups of carbon paper could play some role in augmenting redox activities on its surface, though this is outside of the scope of the present study.


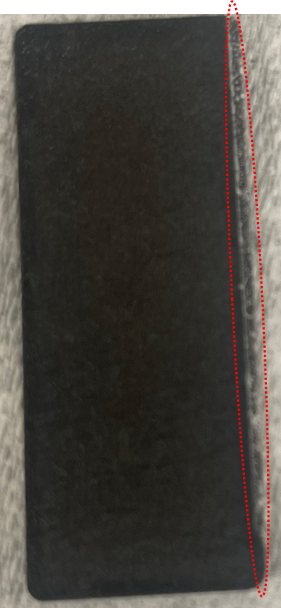


Figure S3. Carbon electrode retrieved from the cycled flow cell. A picture of a disassembled electrode after long-term cycling. The white deposit on the carbon electrode near the inlet is emphasized by a red circle. This deposit could clog the flow pathway as well as reduce the surface area, thus affect the over potential during the long-term cycling.

Figure S4. A symmetric cell using Fe-NTMPA_2_ electrolytes as both catholyte and anolyte. The charge and discharge currents vary from 5 to 100 mA/cm^2^. The black arrow indicates the voltage jump appearing at higher current densities.


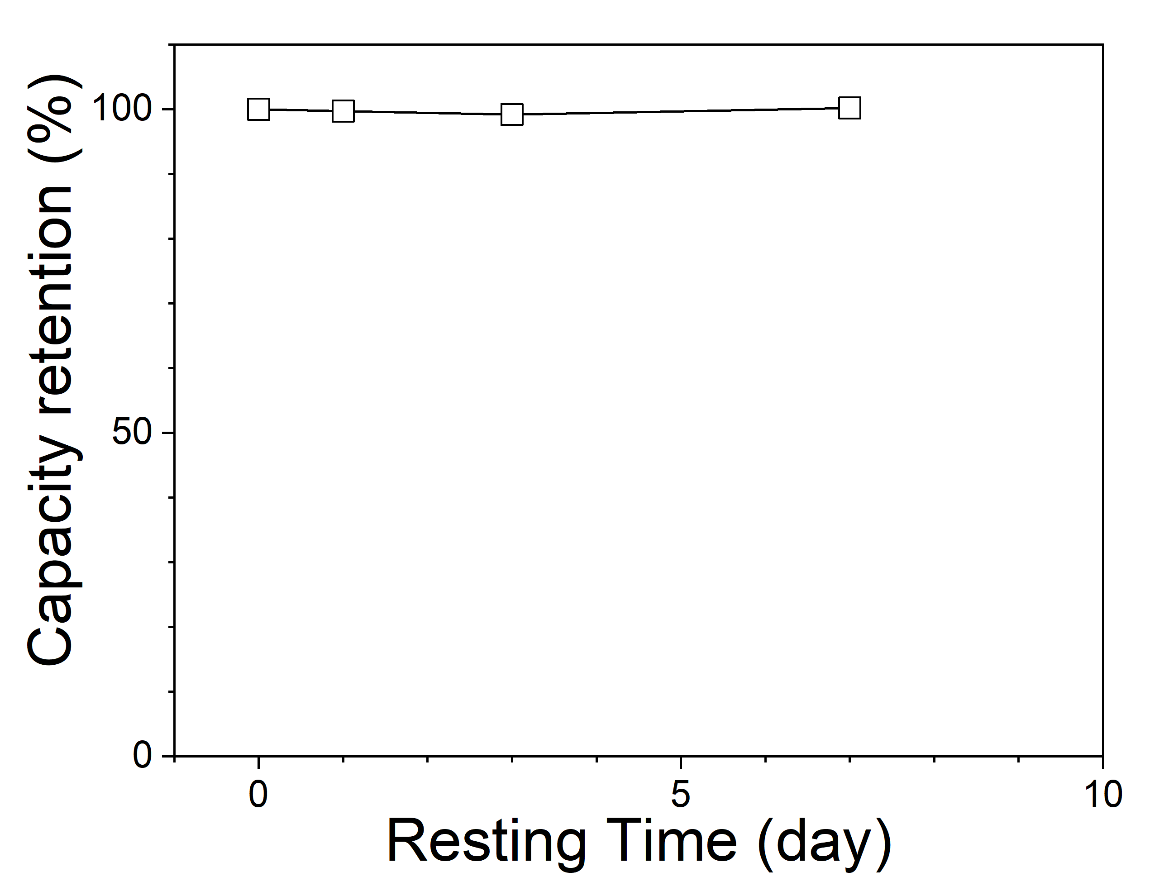


Figure S5. Capacity retention rate while holding the flow battery at 100% SOC over 7 days.

Table S1. Results of linear combination analysis and goodness of fit, *R*-factor (fit range 7115 to 7155 eV). The standards are 100 SOC (Fe^2+^) and 0 SOC (Fe^3+^).

| **Sample** | **100 SOC / Fe^2+^** | **0 SOC / Fe^3+^** | ***R*-factor** |
| --- | --- | --- | --- |
| 25 SOC | 0.28 | 0.72 | 0.0004 |
| 50 SOC | 0.53 | 0.47 | 0.0007 |
| 75 SOC | 0.78 | 0.22 | 0.001 |

(a)

(b)

Figure S6. X-ray absorption experiment. (a) Fitting XANES spectra using 0% SOC and 100% SOC as references. (b) XAS spectra (XANES and EXAFS regions) of Fe K-edge for Fe(ClO_4_)_2_ and Fe-NTMPA_2_.

Table S2. Fe K-edge EXAFS fit parameters Fe(III)-NTMPA_2_ (0 SOC) and Fe(II)-NTMPA_2_ (100 SOC) samples and iron-oxygen (first shell) and iron-phosphorus bond distances.

| **Sample** | **Path** | **CN** | **R (Å)** | **σ^2^ (Å^2^)** | **ΔE_0_ (eV)** | ***R*-factor** |
| --- | --- | --- | --- | --- | --- | --- |
| 100 SOC / Fe^2+^ | Fe-O  Fe-P1  Fe-P2 | 6^f^  2^f^  2^f^ | 2.05 (0.02)  3.16 (0.02)  3.50 (0.06) | 0.011 (0.001)  0.004 (0.002)  0.015 (0.009) | 2.9 (0.6) | 0.02 |
| 0 SOC / Fe^3+^ | Fe-O  Fe-P1  Fe-P2 | 6^f^  4^f^  2^f^ | 2.00 (0.01)  2.68 (0.04)  3.17 (0.03) | 0.009 (0.002)  0.023 (0.008)  0.005 (0.005) | 2.9^f^ | 0.04 |

CN is the coordination number; ^f^ fixed; R is the atomic distance (Å); σ^2^ is the EXAFS Debye-Waller factor (Å^2^); ΔE_0_ is the shift in energy (eV); *R*-factor is the closeness of fit. Fitting k-range from 1.8 to 9.4 Å^-1^; Fourier transform fitting range from 1.2 to 3.6 Å. The amplitude reduction factor S_0_^2^ = 0.965. Number in parenthesis is one SD error.

Table S3: DFT calculated total energy (Δ*E*), zero-point vibrational energy (Δ*ZPVE*), entropy (Δ*ST*), temperature (T=298K) and Gibbs free energy (Δ*G*=Δ*E*+Δ*ZPVE*-Δ*ST*) for the interconversion of S1 and S2 at the B3LYP-D3(BJ)/def2-TZVP level of theory in implicit aqueous solvent.

| **Without N-H** | **Fe(III)** | **Fe(II)** |
| --- | --- | --- |
| **Δ*E*(S1-S2)** | -0.741 | -1.887 |
| **Δ*ZPVE*(S1-S2)** | 0.347 | 0.313 |
| **Δ*ST*(S1-S2)** | -0.123 | -0.125 |
| **Δ*G*(S1-S2)** | -0.271 | -1.449 |

| **With N-H** | **Fe(III)** | **Fe(II)** |
| --- | --- | --- |
| **Δ*E*(S1-S2)** | -0.789 | -1.172 |
| **Δ*ZPVE*(S1-S2)** | 0.191 | 0.218 |
| **Δ*ST*(S1-S2)** | 0.013 | 0.025 |
| **Δ*G*(S1-S2)** | -0.828 | -1.193 |

*Δ*G*(S1-S2) is free energy difference.

Without N-H: no proton on the single amine of Fe(NTMPA)_2_ complex.

With N-H: the single amine of Fe(NTMPA)_2_ complex still has one proton.

The free energy calculations for both models, with or without N-H, indicate a larger difference in free energy between Fe(II)-S1 and Fe(II)-S2 compared to that of Fe(III)-S1 and Fe(III)-S2.
